# Disease

**Published:** 1883-06

**Authors:** W. H. Atkinson


### DISEASE.

BY W. H. ATKINSON, M. D., D. D. S.

READ BEFORE THE DENTAL SOCIETY OF THE STATE OF NEW YORK, AT ALBANY, MAY 10, 1883.

Ease and *dis*-ease are but effects of nutrient movements that may be perceived or unperceived by the body under their dominion, in accord with the attention or inattention of consciousness of the body. Ease (health) is a regulated interchange of activities in molecules in accord with the layout or type of organs. *Dis*-ease is disturbance of this order of inter-blending of mass and energy. Coalescence of these, mass and energy, is the process by which variety in conformation of body is produced and maintained. The degrees of satisfaction of these blendings known as ripeness, or ripening, present us with nebulæ, suns, planets and inhabitants of planets.

To get a complete and exhaustive conception of what disease is, it will be necessary therefore to describe in some way the formation and behavior of all bodies subject to disease or imperfect nutrient activity.

1. The radiance of solar fulness in filling cosmic void, produces planets and inhabitants of planets by a procession of impacts of energy exemplified in the production and feeding (maintaining) of individual bodies.

2. Any disturbances of the succession of impacts minifies or prevents the formation and feeding of these bodies.

3. When bodies or parts of bodies are thus minified, they are weak, and hence they are unable to operate the changes denominated functional activities of molecular, corpuscular, tissual, organic, systemic, or conscious manifestation of character in their completeness. This minification of functional power lays the foundation of unrest, dissatisfaction of demand for energy, which is the condition known as disease.

The way in which this comes about is so occult and complicated as to render it difficult of apprehension and explanation. Radiance penetrating cosmic void produces molecular-mass, from which arise by continuity of the alternate on-going and arrest of radiant impact, the various bodies which appear in the heavens. These consist of solar systems made up of suns, planets and inhabitants of planets.

Radiance is the power, and mass the matrix by which worlds are produced. The process of production may be said to consist of interpenetration, intussesception, invagination and coalescence of these dynamic and static aspects of body and being, in accord to type in molecular, corpuscular, tissual, organic, systemic and conscious manifestation.

It is said that the debris of minerals makes vegetables a possibility, and that the disintegration of these makes the basis of the animal kingdom.

In view of this statement, may we not legitimately accept the saying that in all forms of feeders, the debris of each category becomes the food of the next below it in the organization of the inhabitants of this and other planets? Acceptance of affete, and rejection of effete or excessive portions of food constituting the process of nutrition, must be regular and rythmical to be physiological. When irregular by deficiency or perturbation of the nutrient currents, it becomes pathological—*disease.*

Is disease inherited?

The possibility of both ease (the study of which is physiology) or *dis*-ease (the study of which is pathology) being transmitted from antecedent bodies has been demonstrated.

Man, as the culmination of the manifoldness and simplicity of cosmic and planetary function, is the embodiment of psychic and bodily manifestation of FUNCTION, or demand and supply in the production and maintenance of suns and systems in space, by solar fulness penetrating cosmic void. The begetting, gestating, (ripening) and disrupting of worlds, is represented by the generation and career of inhabitants of planets, at the head of which the human race is conspicuous.

The revelation and ripening of the formulæ of science, speculative and exact, have been obtained through mutations in mind and matter, being grasped by consciousness in multiplied observations. Time and strength would fail us were we to attempt justification of these statements by representing the mutations that mark the history of the development of the sciences.

If science be the "record of the regulated observation of effects," as is so persistently reiterated by materialists, we are bound to ask what "observation" consists of? First, we must have the power of perception; second, this must be so developed as to enable us to correlate observations in understandable series, so as to reveal the principles laws and methods of procedure to consciousness.

As mathematics is independent of bodily or material entanglement by belonging exclusively to the domain of consciousness or mind, we can only bring it to bear upon physics as the means of discovery and correction of the formulæ of science in astronomy, geography and physiology.

A close reading of the record will show this in considering the old and new styles of calculating time. In the discovery of gravitation, the precession of the equinoxes and of planets under the guidance of the imperfect astronomy (astrology) of the ancients, the divisions of time were insufficient to so calculate the seasons of the year as to have them occur in the same months.

By the advent of Copernicus this was corrected, and the new style of measurement of time was promulgated, which still holds good. In pursuit of the explanation of the precession of the equinoxes, the shape of the earth was re-examined as to a possible causative relation to retarded diurnal motion.

It turns out that the equatorial diameter of the globe exceeds that of the polar diameter sufficiently to account for the difference between the old and new styles of estimating, by the difference of the sun's attraction between sphericity of the earth in old style, and an oblate form in new style. The discovery of Neptune was the result of calculations based upon observations of perturbation in the orbits of other planets.

Thus astronomy and geography have progressed and are advancing to the higher and better pronouncement, as well as physiology, psychology and hygiene.

Coalescence (or flowing together) takes place in ethers, gases and liquids or fluids; therefore solids and semi-solids must be melted or dissolved to reduce them to a state capable of intimate admixture or blending. Incomplete coalescence of primates precedes imperfect embodiment of types in molecules, and their massing in cosmic dust—nebula, sun, star, planet, and occupant of planet—and is inception of disease in crystal, cell, corpuscle, organ, and system.

Admixture is aggregation; blending is such a change of molecular constitution as to render heterogeneous masses homogeneous in character. A condition known as temperature is a prominent factor in the conversion of solids and semi-solids into liquids, gases, ethers and radiants; and it must be taken into account in any investigations of healthful or fractional conditions of functioning bodies.

Completeness or wholeness of function is health, or wholeth; while incompleteness of performance of process fractionalizes its career, which is minified function, or *dis*ease. The inception of disease may be said to depend upon perturbation in temperature of body, known as taking cold—"catching cold."

Parallelism of all the stages of the processes of feeding affords normal blood, out of which the tissues are formed and nourished under the fulfillment of the law of demand and supply in every form of functioning body, large or small.

Deficiency (hunger) calls for sufficiency, which is attained by exercise of the whole range of movements, viz: 1, prehension; 2, commutation (mastication); 3, insalivation; 4, deglutition; 5, chymification; 6, emulsification of carbo-hydrates; 7, chylification (precipitation of effete or unchurned portions of food mass); 8, absorption into the blood tract through the chyliferous vessels; and 9, vivification by admixture of oxygen in the respiratory tracts. When this is effected in full measure we have pure blood out of which to sustain the elements of the functioning machinery in health in formed bodies; and producing these elements in forming bodies by converting blood into protoplasm and this into embryonal corpuscles, and these into the tissues and organs of functioning systems, by aggregation, according to typal requirement in the construction and nourishment or support of the parts and the entire system. Let us state, then, that from bathybius (protoplasm) to blood and breath, and from breath to blood and protoplasm (bathybius) we may trace the line of *solar radiancy* as the + (plus) energy that awakens the potencies of atoms and engages them in forming the molecules out of which the proteinaceous compounds are generated that are capable of being metamorphosed into tissues. Let us ask, then, what are the factors of function? And answer by stating, Light is the + (plus) and Atoms the – (minus) cosmic entities, movements in which display to observation cosmic, solar, stellar and planetary systems which are the make-up of the cosmos in the great sense—the universe, as it is called.

Take a complete system of organs at the point of its career known as its beginning; it will be perceived that all the possibilities of function are immanent and emanant in it and its surroundings.

Use of the machinery wears it out by reason of the changes wrought in its parts at their points of contact, under the impact and variety in mode of the energy that operates the functions of the parts and the entire machine. This waste of wear, or loss' must be restored to it by repair of the machine to keep it in working order. This process of repair in functioning bodies is known as feeding; and is operated by a complicated series of preparatory sub-processes of a triple apparatus in respiratory, circulatory and alimentary tracts. These sub-processes operate upon the crude material out of which pabulum is manufactured, from which tissues are fed by appropriation of affete and rejection of effete and excessive portions of pabulum. Appropriation of pabulum is called assimilation, and is only attributed to the vegetable and animal kingdoms in extant physiologies.

This process of appropriation and rejection of portions of pabulum involves fine division and admixture of materials of the food in the crude sense. In the mineral kingdom we would say solution and precipitation were involved in the production of crystals. In a solution of silicon, precipitation produces *silex*, by crystalization according to a special six-sided type; and *silica* in an amorphous powder.

This immanance of type is always a factor in building and destroying mineral, vegetable and animal bodies.

Mineralistic parts of vegetables constitute their solidity in stalk, fruit and seed.

These elements are carried to place by solution in water through channels of circulation for such fluids.

Water is called the "universal solvent," and the saying finds confirmation in all physiological investigations.

An English experimentalist finds that for every pound of mineral matter assimilated by a plant an average of 2,000 pounds of water is absorbed.

At the French Observatory of Mt. Souris it was found that in rich soil 727 pounds of water passed through the roots of wheat plants for every pound of grain produced; while in a very poor soil

2,693 pounds of water passed through the wheat roots for each pound of grain matured.

The depositing of these solids necessitates a dissipation of the water, which is effected by transpiration, to form the halm, fruit and seed. This is purgation (expurgation) in the vegetable domain.

The lowest form of animals so nearly repeat this mode of appropriation of weak solution of pabulum as to make it difficult to detect the difference in mode of assimilation.

The amœbæ bouche and debouche at the same point of their bodies.

That is, they improvise mouth and anus on the side of their bodies next the thing they wish to devour, and thus eat by closing around the article of food and defecate by unfoldment of the body walls, allowing the unassimilable parts of food to fall away. This is the earliest example of the division of animals, classified as monotremata, thus showing us that vomiting and purging are identical in purpose, viz.: getting rid of debris and excess of food.

Those conditions known as *diseases* are the results of perturbation in the nutrient processes, which results have not been eliminated in time to prevent debility, aberration or death of the elements of tissues. Thus local disease is ever the manifestation of constitutional debility being focalized by local lesions that would be of no account in soundness of blood crasis, i. e., all sorts of fracture heal by first intention, by simple reposition of the divided parts in healthy bodies; while tubercle, tumor, cancer and infectious diseases hold high carnival in the imperfectly elaborated blood plasm.

To prevent disease, live in accordance with hygienic laws, the principal of which is the intelligent use of general gymastics, thus washing out all the channels of nutrient activity by vigorous exercise.

For special obstructions resort to special gymnastics, namely, in respiratory tracts by vigorously breathing, in vascular tracts by accelerated circulation, thus hastening secretion and excretion, and in alimentary tracts by vomiting, purging and sweating.

For infectious diseases, neutralize the poison, and for local disease extripate the abnormal part.

